# A novel approach to resilience and its links with education and Alzheimer's disease genetics

**DOI:** 10.1002/alz.70379

**Published:** 2025-07-04

**Authors:** Maria Carrigan, Diana I. Bocancea, Jacob Vogel, Anna C. van Loenhoud, Niccoló Tesi, Frederik Barkhof, Paul J. Lucassen, Wiesje M. van der Flier, Harm J. Krugers, Sven J. Van der Lee, Rik Ossenkoppele

**Affiliations:** ^1^ Alzheimer Center Amsterdam, Neurology, Vrije Universiteit Amsterdam Amsterdam UMC location VUmc Amsterdam the Netherlands; ^2^ Amsterdam Neuroscience, Neurodegeneration Amsterdam the Netherlands; ^3^ Faculty of Science Swammerdam Institute for Life Sciences University of Amsterdam Amsterdam the Netherlands; ^4^ Department of Clinical Sciences Malmö Faculty of Medicine SciLifLab, Lund University Lund Sweden; ^5^ Section Genomics of Neurodegenerative Diseases and Aging, Department of Clinical Genetics Vrije Universiteit Amsterdam, Amsterdam UMC Amsterdam the Netherlands; ^6^ Delft Bioinformatics Lab Delft University of Technology Delft the Netherlands; ^7^ Queen Square Institute of Neurology and Centre for Medical Image Computing University College London Queen Square London UK; ^8^ Department of Radiology & Nuclear Medicine Amsterdam Neuroscience, Vrije Universiteit Amsterdam, Amsterdam UMC Amsterdam the Netherlands; ^9^ Department of Epidemiology & Data Science Amsterdam UMC location Vrije Universiteit Amsterdam Amsterdam the Netherlands

**Keywords:** Alzheimer's disease, canonical correlation analysis, cognitive resilience, genetic risk factors, global cognition, magnetic resonance imaging

## Abstract

**INTRODUCTION:**

Cognitive resilience refers to maintaining cognitive function despite Alzheimer's disease (AD) pathophysiology.

**METHODS:**

We analyzed amyloid‐positive individuals across clinical stages of AD in two cohorts: the Amsterdam Dementia Cohort (ADC, *N* = 1036) and Alzheimer's Disease Neuroimaging Initiative (ADNI, *N* = 685). Cognitive resilience was conceptualized from a canonical correlation analysis of magnetic resonance imaging and neuropsychological data in each cohort separately. Model validation involved education as a resilience proxy and key genetic factors (apolipoprotein E [*APOE*] ε4 and *APOE* ε2) of AD. We explored associations between 83 AD risk loci and cognitive resilience.

**RESULTS:**

Resilience was correlated with education (ADC: β = 0.144, *p* < 0.001; ADNI: β = 0.149, *p* < 0.001) and *APOE* ε4 (β_meta‐analysis _= –0.052, *p* = 0.014). Exploratory single nucleotide polymorphism meta‐analysis identified potential involvement of genetic variants around genes *UNC5CL*, *USP6NL*, and *TPCN1* in lower, and genes *COX7C* and *MINDY2* in higher resilience.

**DISCUSSION:**

Our novel resilience approach showed conceptual validity and potential for future discovery of resilience‐related genetic variants.

**Highlights:**

· We define a novel approach to resilience using canonical correlation analysis (CCA).
· Apolipoprotein E ε4 is linked to lower resilience, suggesting increased vulnerability.
· Genetic loci around *COX7C* and *MINDY2* are potentially involved in higher resilience.
· This novel approach may be used for multi‐cohort studies such as genome‐wide association studies in the future.

## 1 BACKGROUND

Aggregation of amyloid beta (Aβ) is among the earliest pathological changes in Alzheimer's disease (AD).^1^ Despite abundant pathology, some individuals show limited decline in memory and other cognitive functions. Such individual differences in AD trajectories suggest the presence of protective mechanisms, a concept known as cognitive resilience.^2^

Resilience to AD‐related neurodegeneration is influenced by various factors, including age, sex, genetic factors, environment, and lifestyle.^3^ With consensus on AD‐causative mutations in genes *PSEN1, PSEN2*, and *APP*, involvement of genetic factors in AD is well established and heritability is estimated around 60% to 80%.^4^, ^5^ In sporadic AD, the apolipoprotein E (*APOE*) ε4 variant raises the risk of developing AD by three to fifteen times,^6^, ^7^ and a further 83 AD‐related genetic loci have been identified in a recent genome‐wide association study (GWAS).^5^ Conversely, *APOE* ε2 and the recently discovered *APOE* ε3 Christchurch mutation delay or even protect against the development of clinical symptoms.^3^, ^6^, ^8^ This discovery of protective genes to AD sparked growing interest in genetic variants that may boost cognitive resilience.^3^, ^4^, ^9^, ^10^, ^11^

In the past decade, research on cognitive resilience has relied on quantifying this concept using proxy variables (e.g., education, IQ) or, more recently, various residual approaches. Residual measures are derived from regression models of cognition on brain pathology (e.g., tau or atrophy) from which the model error term constitutes the measure of resilience.^12^ As such, residuals reflect the degree of cognitive resilience based on observed versus predicted cognition scores relative to the pathological burden.^11^, ^13^ While these measures are good predictors of future cognitive decline,^14^, ^15^ they constitute a derivative two‐step approach in which the residual is first calculated from the regression model and subsequently subjected to the analysis of interest as its own measure of resilience. This can lead to confusion in interpretation and understanding of the etiology of cognitive resilience. To resolve this issue while maintaining the conceptual advantages of the standard residual approach, we propose a novel approach to operationalizing cognitive resilience by using a canonical correlation analysis (CCA) model.^16^ CCA is a multivariate statistical model that identifies associations between two high‐dimensional sets of variables, in this case cognition and magnetic resonance imaging (MRI) data. Specifically, CCA finds the shared correlation structure within the co‐analyzed variable sets and describes the symmetrical linear relationship that summarizes the data compactly. CCA has been applied previously to study brain–behavior relationships,^17^, ^18^, ^19^ demonstrating its suitability in summarizing multidimensional data into latent dimensions.^16^ CCA's main advantage over the standard residual approach is its ability to leverage all available data across domains, rather than focusing on select variables. This approach effectively captures the complexity of cognition and neurodegeneration when pooling data from multiple cohorts, making it particularly useful for large‐scale multicenter GWAS.

To provide proof‐of‐principle for this approach, we defined a CCA‐based quantitative framework of resilience using cognition and neuroimaging data from two independent cohorts of Aβ‐positive individuals. We first validated the model by examining the association between our resilience framework and education (an established resilience proxy). Second, we investigated the association of key genetic AD risk (i.e., *APOE* ε4) and AD protective (i.e., *APOE* ε2) factors with resilience. Third, we explored the association of cognitive resilience on a single nucleotide polymorphism (SNP) level of 83 GWAS‐based AD genetic loci.^5^ We hypothesized to observe a positive association between education and resilience, and a negative association between *APOE* ε4 and resilience. Because the protective effects of *APOE* ε2 are primarily linked to Aβ aggregation, we anticipated no association between resilience and *APOE* ε2 in either cohort due to our sample selection. Finally, based on incomplete annotation of functional downstream effects of genetic loci associated with risk for developing AD, we hypothesized to discover associations between some of these variants and cognitive resilience.

## 2 METHODS

### 2.1 Sample characteristics

#### 2.1.1 Amsterdam Dementia Cohort

We inlcuded a total of 1036 Aβ‐positive subjects across clinical stages of AD from the Amsterdam Dementia Cohort (ADC, Table 1) who were assessed at the memory clinic between 2002 and 2020. Participant selection was based on the following criteria: (1) Aβ positivity as determined using either cerebrospinal fluid (CSF; Aβ_42 _< 813 pg/L^20^ [*n* = 1059]) or positron emission tomography (PET; i.e., [^11^C]Pittsburgh compound‐B or [^18^F]flutemetamol^21^ [*n* = 88]), (2) availability of a MRI scan on 1.5 or 3T scanner, (3) availability of neuropsychological data, and (4) availability of genetic data. We investigated subjects across clinical stages of AD, including cognitively unimpaired (CU) individuals (*n* = 112), those with mild cognitive impairment (MCI, *n* = 219), and individuals with AD dementia (*n* = 705). Clinical diagnosis was established in a multidisciplinary team by consensus, according to the respective criteria by the National Institute on Aging–Alzheimer's Association.^22^ Individuals who presented with subjective cognitive complaints but tested within normal limits at neuropsychological examination were classified as CU, as were those with no complaints but Aβ positivity.^23^ Exclusion criteria were: (1) substantial scanning or movement artifacts on MRI as well as a failed image processing scan; (2) an interval > 6 months between MRI and neuropsychological testing in AD cases, and > 12 months in CU or MCI cases; or (3) age < 50 years. All participants underwent standard dementia screening, including a structured caregiver interview, medical history and physical examination, lumbar puncture and/or PET imaging, brain MRI, genotyping, and extensive neuropsychological testing.^24^ In addition, as a measure of education, participants were scored using the seven‐item Verhage scale, which is a standardized measure based on the Dutch educational system, with higher scores representing more advanced levels of education (e.g., 1 = primary school not completed, 7 = academic degree).^25^ For comparability to the Alzheimer's Disease Neuroimaging Initiative (ADNI), we converted the measure of education in ADC from Verhage to years (e.g., 1 = 5 years, 7 = 17 years; Table S1 in supporting information).^26^

**RESEARCH IN CONTEXT**

1. **Systematic review**: Literature search revealed that several studies have examined the biological and genetic underpinnings of cognitive resilience in the context of Alzheimer's disease (AD), but there has been limited consensus on methodologies for assessing resilience. This study contributes to the field by proposing a canonical correlation analysis to investigate cognitive resilience in amyloid‐positive individuals across two cohorts: the Amsterdam Dementia Cohort and Alzheimer's Disease Neuroimaging Initiative.
2. **Interpretation**: The study found resilience to correlate with education and apolipoprotein E ε4. In addition, we identified several single nucleotide polymorphisms to be potentially involved in the mechanisms behind resilience, including genetic variants around genes *TPCN1, UNC5CL, USP6NL*, *COX7C*, and *MINDY2*. Our findings highlight the effectiveness of canonical correlation analysis in measuring resilience, particularly in multi‐cohort studies.
3. **Future directions**: Future research should include bigger samples to explore the genetic mechanisms of resilience, focusing on sex‐dependent variants, and investigate the role of lifestyle factors in enhancing resilience.

**TABLE 1:** Sample characteristics.

	ADC (*N* = 1036)	ADNI (*N* = 685)
Sex		
Males (%)	525 (50.67)	376 (54.91)
Age (years)		
Mean (SD)	65.9 (6.80)	75.0 (7.25)
Diagnosis		
AD	705 (68.1%)	212 (30.9%)
MCI	219 (21.1%)	286 (41.8%)
CU	112 (10.8%)	187 (27.3%)
*APOE*, carriership		
ε4	726 (70.1%)	447 (65.3%)
ε2	62 (6.0%)	35 (5.1%)
Education (years)		
Mean (SD)	11.5 (2.97)	16.0 (2.79)
MMSE		
Mean (SD)	22.6 (5.37)	26.2 (3.76)
MRI AD signature^a^		
Mean (SD)	2.63 (0.151)	2.64 (0.190)

Abbreviations: AD, Alzheimer's disease; ADC, Amsterdam Dementia Cohort; ADNI, Alzheimer's Disease Neuroimaging Initiative; *APOE*, apolipoprotein E; CU, cognitively unimpaired; MCI, mild cognitive impairment; MMSE, Mini‐Mental State Examination; MRI, magnetic resonance imaging; SD, standard deviation.

^a^ Cortical thickness (mm).

#### 2.1.2 ADNI

We further assessed 685 Aβ‐positive individuals across clinical stages of AD from the ADNI cohort, included between 2006 and 2020 (Table 1). Aβ positivity was defined using cohort‐specific PET standardized uptake value ratio (SUVR) thresholds (i.e., [^11^C]Pittsburgh compound‐B [1.5 SUVR], [^18^F]Florbetapir [1.11 SUVR] or [^18^F]Florbetaben [1.08 SUVR]).^27^, ^28^ Specifically, we used demographic data including education as measured in years, neuropsychological data, as well as MRI and genetic data. Detailed information on methodology of either of these data sets can be found on www.adni‐info.org. Participant selection was based on the same criteria as used in ADC. Our sample consisted of CU individuals (*n* = 187), individuals with MCI (*n* = 286), and individuals with AD dementia (*n* = 212).

### 2.2 Measures of cognition

#### 2.2.1 ADC

Cognitive performance was sampled from several previously established cognitive domains^23^ using a comprehensive battery of neuropsychological tests. Global cognition was assessed using the Mini‐Mental State Examination (MMSE). We included total immediate and delayed recall of the 15 words test (a Dutch version of the Rey Auditory Verbal Learning Test [RAVLT]), the recognition index calculated based on false positives and false negatives of the 15 words test, and total recall on condition A of the Visual Association Test as measures pertaining to the memory domain. The executive functioning domain was covered by the Frontal Assessment Battery, digit span backward, Stroop test condition 3 (color‐word task), Trail Making Test (TMT) Part B (TMT‐B), and letter fluency. For the attention domain, digit span forward, TMT Part A (TMT‐A), and Stroop test conditions 1 and 2 (word and color, respectively) were used. Finally, we included the naming part of the Visual Association Test and Animal Fluency to measure the language domain. For individuals with missing TMT‐B data but available data for the TMT‐A, we imputed TMT‐B by calculating the missing score using the available individual TMT‐A score and a diagnostic‐group specific TMT‐A/TMT‐B ratio. We excluded individuals (*n* = 8) with fewer than three cognitive tests available. Further, we excluded those cognitive tests that were missing in > 50% of the participants (e.g., the Complex Figure of Rey that was only available in 34.44% of participants), resulting in a total of 16 cognitive variables. Finally, the Stroop test and TMT scores were inverted to match the directionality of the other 11 cognitive tests (i.e., lower scores representing worse cognitive functioning) to facilitate interpretation of the results.

#### 2.2.2 ADNI

A total of 11 neuropsychological tests were included from ADNI, covering five cognitive domains in addition to global cognition, which was assessed using the MMSE. The memory domain was measured by the RAVLT immediate and delayed recall, Logical Memory I, as well as the Alzheimer's Disease Assessment Scale Cognitive subscale (ADAS‐Cog) immediate and delayed recall. As in ADC, the TMT‐B was included as a measure of executive functioning, while Part A of the same test was used to assess the attention domain. For the language domain, we included Animal Fluency and the Boston Naming Test. Finally, we included the Clock Drawing Test to assess visuospatial capabilities. Individual test scores were further processed for the CCA model. Missing data on TMT‐B was imputed similarly to the ADC cohort, and similar exclusion criteria were applied. In further concordance with procedures performed in ADC, TMT scores were inverted, as were scores of the RAVLT delayed recall, and ADAS‐Cog immediate and delayed recall.

### 2.3 MRI acquisition and pre‐processing

#### 2.3.1 ADC

Acquisition of 3D T1‐weighted MRI was performed on 10 different MRI scanners of 1.5–3T (*n* = 188 and *n* = 959, respectively) according to standardized acquisition protocols as described elsewhere.^29^ The MRI data was processed with FreeSurfer software (http://surfer.nmr.mgh. harvard.edu/; v7.1). We extracted cortical thickness and surface area in 68 whole‐brain segmented regions of the Desikan–Killiany atlas,^30^ as well as 16 subcortical volumes, intracranial volume (ICV), and white matter hypointensities (WM‐hypo; a T1‐image derived proxy measure of white matter hyperintensities).^31^, ^32^, ^33^ The MRI data was quality checked (QC) visually, excluding scans with poor cortical segmentation while poorly segmented subcortical volumes were set to missing values at the regional level. Where longitudinal MRI data was available, we selected the scan closest to the date of neuropsychological testing, but no more than 6 months apart in individuals with AD and no more than 12 months apart in CU individuals. Cortical thickness measures, surface areas, and subcortical volumes were harmonized using *neuroCombat*,^34^, ^35^ including scanner type as a nuisance variable and the biological variables age, sex, and diagnosis as variables whose explained variance was preserved in the data. Subsequently, we calculated weighted cortical thickness composites for whole brain, frontal, temporal, parietal, and occipital regions, and an unweighted cortical thickness composite for AD‐signature regions. WM‐hypo scores were inverted to align the directionality among the MRI variables (i.e., higher values reflect a positive outcome across all variables) and log‐transformed. All volumetric variables (i.e., subcortical volumes and WM‐hypo) were adjusted for ICV by regressing out the effect of ICV from each variable. This adjustment was performed after imputing missing values, as described below.

#### 2.3.2 ADNI

We assessed MRI data from a total of 21 different MRI scanners of 1.5–3T (*n* = 235 and *n* = 450, respectively). ADNI images were downloaded and preprocessed in house using the same FreeSurfer pipeline used in ADC. As these images were not visually QCed, they were processed as follows. We identified potentially failed segmentations based on the *SurfaceHoles* variable (i.e., scans with a value larger than median ± 3 x interquartile range thresholded *SurfaceHoles*, given that a larger value has been suggested as an indicator of poor segmentation quality) and assessed respective images visually. For subcortical volumes, we identified outlying values (based on the diagnostic group–specific sum of the mean ± 2 standard deviations) and subsequently set those to missing values. All further data processing was performed according to procedures employed in the ADC, extracting all MRI variables described above.

### 2.4 Cognitive resilience

We define the cognitive resilience framework as the variance in cognition unexplained by brain atrophy in participants with amyloid pathology, using the first pair of canonical variates from CCA to quantify cognition and brain dimensions (see section 2.6 for details). While this approach uses the same underlying conceptual framework as a residual method, the difference is that rather than extracting the model error term (i.e., residuals) and calling it cognitive resilience, we maintain the latent variables of cognition and brain as they are estimated from the CCA and incorporate them in the following analyses (i.e., tests for associations with genetic variables) within one model (as explained below).

### 2.5 Genetic profiling/measures

#### 2.5.1 ADC

Genetic variants were determined using standard genotyping, imputation, and QC methods as described elsewhere.^36^, ^37^ In essence, high‐quality genotyping in all individuals was performed using Illumina Global Screening Array (GSA; Illumina, Incl; individual call rate  > 99%, variant call rate  > 99%). All individuals’ genetic sex matched their reported sex. We excluded variants deviating from Hardy–Weinberg equilibrium at *p* < 1×10^− 6^, genotypes were lifted over to GRCh38/hg38, and prepared for imputation using provided scripts (HRC‐1000G‐check‐bim.pl) specifying TOPMED as reference panel.^38^ This script compares variant ID, strand, and allele frequencies to the TOPMED reference panel (version r2, *N* = 194,512 haplotypes from *N* = 97,256 individuals).^39^ All individuals and variants were imputed using the Michigan Imputation server (https://imputation.biodatacatalyst.nhlbi.nih.gov/). The server uses EAGLE (v2.4) to phase data and Minimac4 to perform genotype imputation to the reference panel (version r2).^40^, ^41^ Before analysis, we excluded individuals with a family relation (identity‐by‐descent ≥ 0.2),^42^ and we kept only individuals of European ancestry (based on 1000Genomes clustering).^43^ Finally, we extracted imputed dosages of 83 SNPs previously associated with AD. All genetic loci were coded ordinal for further analysis, with 0 = no allele, 1 = 1 allele, and 2 = 2 copies of the respective allele. In addition, for the purpose of this study, all genetic loci were flipped to risk, meaning that the higher the number of alleles the higher the risk of developing AD.

#### 2.5.2 ADNI

Genetic variants were determined by standard genotyping and imputation methods. All individuals were genotyped in three batches as previously described.^44^ We applied established QC and imputation methods to each batch individually. High‐quality genotypes were used in all individuals (individual call rate > 99%, variant call rate > 99%), and departure from Hardy–Weinberg equilibrium was considered significant at *p* < 1 × 10^−6^. Genotypes were then lifted over to GRCh38/hg38, prepared for imputation, and imputed as previously done for ADC individuals. Again, we extracted imputed dosages of 83 SNPs previously associated with AD. Of these, 81 were imputed with high quality, while 2 SNPs were missing (rs117618017 and rs7157106). To increase comparability to ADC, we replaced the missing SNPs by a variant in linkage disequilibrium (rs75763893 and rs2753568, respectively). In addition, due to a low call rate, one SNP (rs56407236) was excluded from further analyses.

### 2.6 Statistical analyses

All data pre‐processing and statistical analysis was performed in R version 4.2.1.^45^

#### 2.6.1 Data imputation

Missing values in the cognitive tests were imputed with a predictive mean matching model implemented with the *mice* ^46^ package. Age, sex, education, diagnosis, and all the available cognitive tests were included in the single imputation model. Similarly, missing values in subcortical MRI volumes were imputed under the same method, using sex, age, diagnosis, the volume variables, as well as the cortical thickness in lobar composite regions, WM‐hypo, and ICV.

#### 2.6.2 CCA

We fitted a CCA model^16^, ^18^, ^47^ to identify latent dimensions of the brain–cognition relationship using the *candisc* package. The model takes as input two data matrices: the X matrix consisting of MRI‐derived brain variables (i.e., all the regional cortical thickness variables, subcortical volumes, WM‐hypo and ICV [1036 × 84]), and the Y matrix of all cognitive tests [1036 × 16] (in ADC, [685 × 84] and [685 × 11] in ADNI, respectively). All variables were *z* scored prior to model fitting using the entire study population as the reference group. The CCA model then decomposes the data into pairs of canonical variates by finding a linear combination of X (MRI) that maximally correlates with a linear combination of Y (cognition). These linear combinations of X and Y constitute the MRI and cognition canonical variates, respectively. The first pair of canonical variates is computed such that their correlation is maximized (i.e., the canonical correlation). Multiple subsequent canonical pairs of brain and cognition variates (orthogonal with all the previous ones) can be calculated, with the maximum number of pairs bound by the dimension of the smallest matrix (16 cognitive tests in ADC). A property of the CCA is that the first X canonical variate explains the largest amount of variance in Y, with each subsequent variate explaining less and less variance in Y. We calculated therefore the amount (i.e., percentage) of variance that each canonical variate explains of its counterpart original variable's data matrix (also called the redundancies).

While we inspected the first three pairs for better understanding of the CCA model output, our interest lies in the first canonical pair, capturing the most percentage of co‐variance and constituting our framework of resilience, hence we report results of this pair further. Significance of the canonical correlations was tested with a permutation test, in which the rows of the Y matrix were shuffled in 10,000 iterations to generate a null distribution of canonical correlations. Furthermore, we extracted and plotted the loadings (i.e., the correlation) of each set of original variables on their respective canonical variates, to investigate the contribution of the individual original variables to the canonical pair. Sex and age were not included in the CCA model and the input variables were not pre‐adjusted for it, as we did not want to remove variance associated with these two variables at this stage. Both age and sex are accounted for in all main models as covariates.

#### 2.6.3 Statistical models

In each cohort separately, we used the first set of canonical variates as our operationalization of resilience for further analyses. First, we tested the association of the canonical variate of cognition with education in a linear regression analysis while adding the canonical variate of MRI, corrected by age and sex.

(1) $$\;Y_{1}\;∼\;X_{1}+Age+Sex+Education$$

Second, we tested the associations of our framework with AD risk (i.e., *APOE* ε4) and AD protective (i.e., *APOE* ε2) factors in separate linear regression analyses correcting for X_1_, age, and sex.

(2) $$\;Y_{1}\;∼\;X_{1}+Age+Sex+APOE_{i}$$

Next, we explored the association of resilience with the most robust genetic loci of AD^5^ on a single‐SNP level in separate linear regression analyses correcting for X_1_, age, sex, and population substructure (PC1–PC5).

(3) $$Y_{1}\;∼\;X_{1}+Age+Sex+SNP_{n}+PC_{1}+PC_{2}+PC_{3}+PC_{4}+PC_{5}$$

We meta‐analyzed the results of the SNPs across both cohorts using a fixed effect inverse variance meta‐analysis.

Finally, given recent recommendations to use interactions to capture resilience,^15^, ^48^ we performed sensitivity analyses using interactions between $X_{1}$ and education, *APOE*, and single‐SNPs.

## 3 RESULTS

### 3.1 CCA model

**FIGURE 1:**
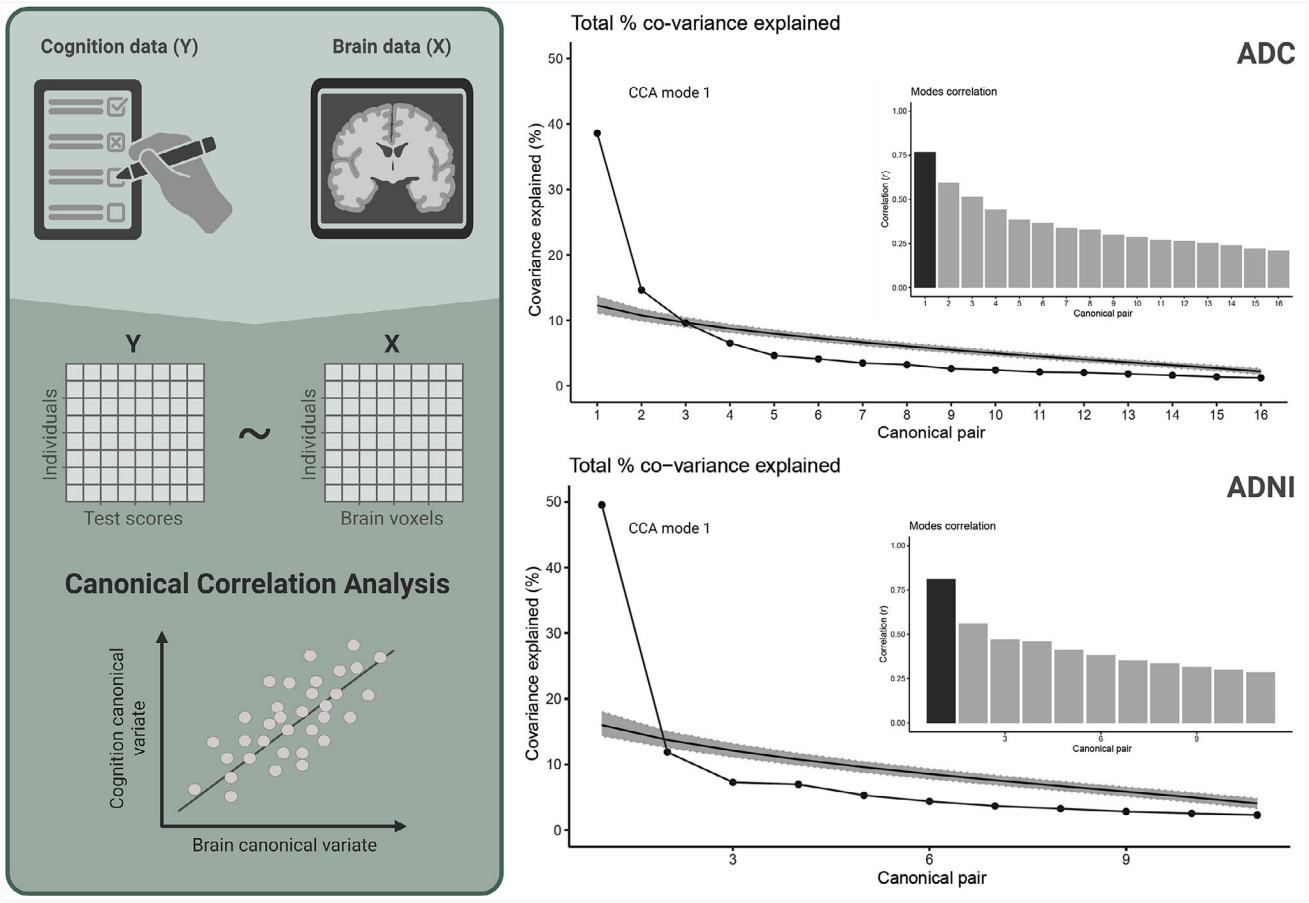
Operationalization of resilience using a canonical correlation analysis. This figure illustrates schematically (left panel) how cognition and MRI data acquired as multidimensional datasets are correlated in a canonical correlation analysis. The right panel show the results of this analysis for the ADC (top) and the ADNI cohort (bottom). We show the percentage of the total covariance in the data explained by each canonical mode, as well as each mode's (i.e., canonical pair of covariates) correlation. Note that the number of modes per cohort is based on the number of cognitive tests included in each model, as this represents the smallest input variable (16 in ADC and 11 in ADNI). ADC, Amsterdam Dementia Cohort; ADNI, Alzheimer's Disease Neuroimaging Initiative; CCA, canonical correlation analysis; MRI, magnetic resonance imaging.

**FIGURE 2:**
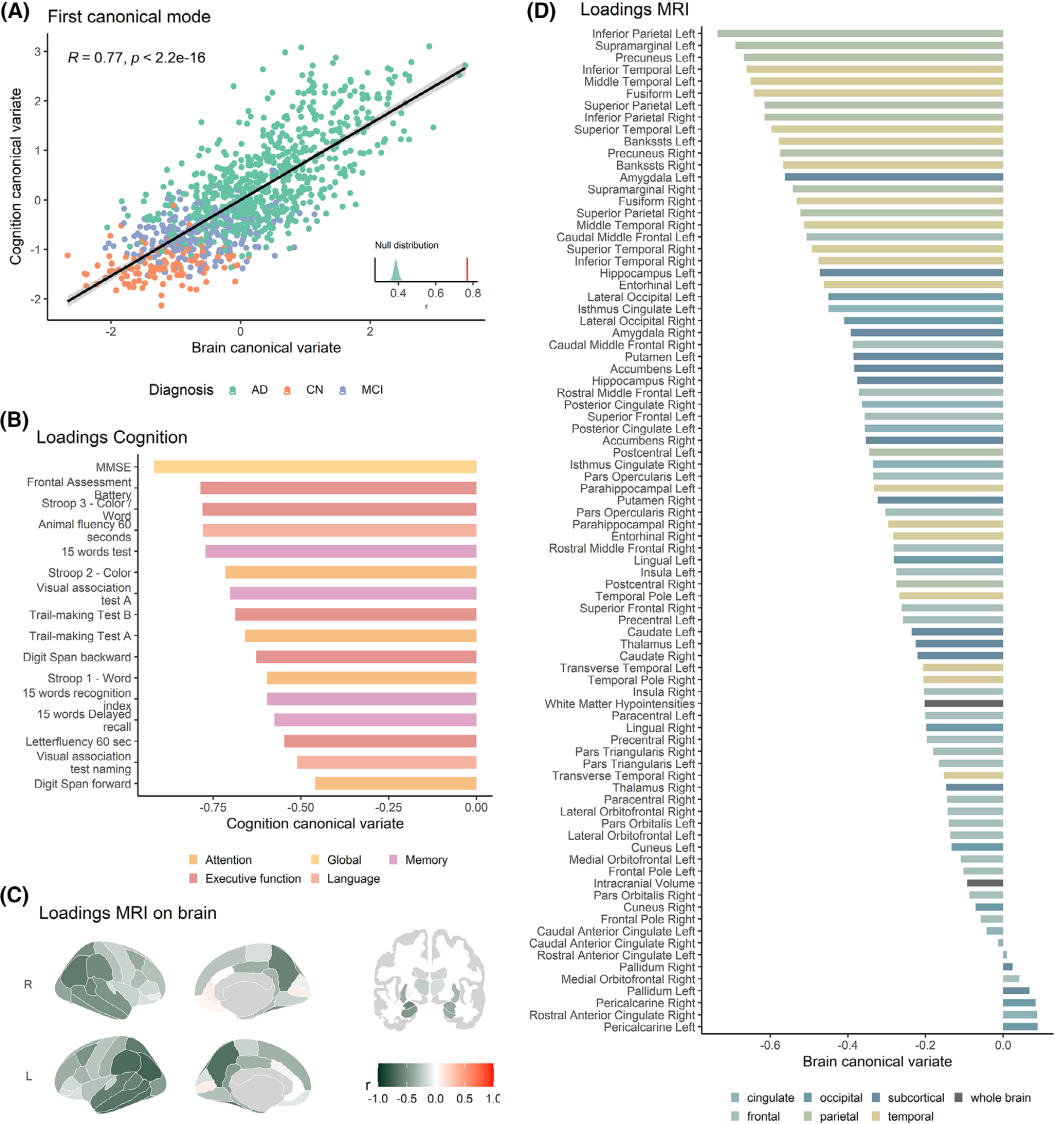
First mode of the canonical correlation analysis in the Amsterdam Dementia Cohort. In (A), the correlation of the first canonical pair is illustrated, colored by diagnosis (cognitively unimpaired, mild cognitive impairment, and Alzheimer's disease dementia) including the null distribution of the permutation test. B, Individual cognitive test loadings on the first canonical variate of cognition (Y), colored by representative cognitive domains. C, MRI variable loadings on the first canonical variate of brain status (X) plotted on the brain, whereas (D) shows the individual variable loadings, colored by brain lobar areas. AD, Alzheimer's disease; CN, cognitively normal; MCI, mild cognitive impairment; MRI, magnetic resonance imaging.

In this section, we present the results of applying the CCA model to two high‐dimensional datasets, including cognition and MRI data. In ADC, given the lowest dimension of 16 cognitive tests, the model yielded 16 modes of association that describe the explained co‐variance. We focus on the first mode in all subsequent analyses, which explained 38.6% of the co‐variance (Figure 1). Figure 2A shows the correlation plot of the first set of MRI versus cognition canonical variates (*R *= 0.77, *p* < 0.001). Figure 2B and 2C show the canonical loadings of each cognitive test on the first canonical variate of cognition (Figure 2B) and the canonical loadings of each MRI variable on the first canonical variate of MRI (Figure 2C). In ADNI, an independent CCA model yielded 11 modes based on the availability of 11 cognitive tests. Comparable to ADC, the first mode showed a correlation of *R *= 0.81 (*p* < 0.001), explaining 49.5% of the co‐variance in the data (see Figure S1 in supporting information). It is important to clarify that these percentages represent the explained co‐variance in the original MRI cognition datasets and not the variance in cognition explained by MRI data (i.e., redundancy). For reference, the variance in cognition explained by MRI (redundancy) in the first mode was 27.3% for ADC and 34.7% for ADNI, highlighting the need to distinguish between these two metrics in interpretation.

Visual assessment of the original variables’ loadings on the canonical variates reveals highly similar pattern across cohorts, indicating that the first canonical dimension of MRI data captures a comparable dimension in the two cohorts (see also Figure S2 in supporting information). In both cohorts, parietal, temporal, and subcortical brain regions loaded most strongly, while frontal, occipital, and cingulate regions loaded less strongly. Despite varying cognitive tests per cohort and the resulting increase in disparity of loadings on the canonical variate of cognition between cohorts, there was a strong overlap regarding the weight of domains. Global cognition as measured by the MMSE loaded very high in both cohorts, while the domain of attention weighed less on the canonical variate of cognition. Memory had a stronger overall loading in ADC compared to ADNI, whereas executive functioning had a higher loading in ADNI.

In sum, the canonical variates captured relatively comparable dimensions of MRI and cognition in two different cohorts characterized by inherent differences in cognitive tests, MRI scanners, and sample compositions.

### 3.2 Education attainment

**FIGURE 3:**
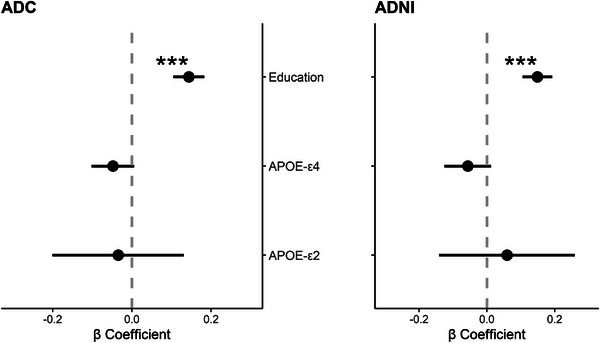
Associations of cognitive resilience with education and *APOE* per cohort. Coefficients of linear regression analyses per cohort between the canonical variate for cognition (Y) and the variable of interest, corrected by the canonical variate for MRI (X), age, and sex. Significant associations between education and higher resilience in both ADC (*p* < 0.001) and ADNI (*p* < 0.001). Non‐significant association for *APOE* ε4 with lower resilience in ADC (*p* = 0.080) and ADNI (*p* = 0.109). No association was found for *APOE* ε2 in either cohort (ADC: *p* = 0.681; ADNI: *p* = 0.562). ADC, Amsterdam Dementia Cohort; ADNI, Alzheimer's Disease Neuroimaging Initiative; *APOE*, apolipoprotein E; MRI, magnetic resonance imaging.

To validate our proposed resilience framework, we tested the association between our resilience framework with education in each cohort separately (Figure 3). We found that higher education was associated with higher cognitive resilience in both ADC (β = 0.144, confidence interval [CI: 0.105, 0.183], *p* < 0.001) and ADNI (β = 0.149, CI [0.105, 0.193], *p* < 0.001).

### 3.3 *APOE* ε4 and *APOE* ε2

Next, we investigated the relationship between the proposed resilience framework and genetic risk (Figure 3). Carriership of the *APOE* ε4 allele showed a non‐significant association with lower cognitive resilience in ADC (β = –0.048, CI [–0.102, 0.006], *p* = 0.080) and ADNI (β = –0.056, CI [–0.125, 0.013], *p* = 0.109). We found no significant relationship between *APOE* ε2 allele carriership and cognitive resilience in either cohort (ADC: β = –0.035, CI [–0.201, 0.131], *p* = 0.681; ADNI: β = 0.059, CI [–0.141, 0.259], *p* = 0.562).

### 3.4 Exploratory single‐SNP level meta‐analysis

**FIGURE 4:**
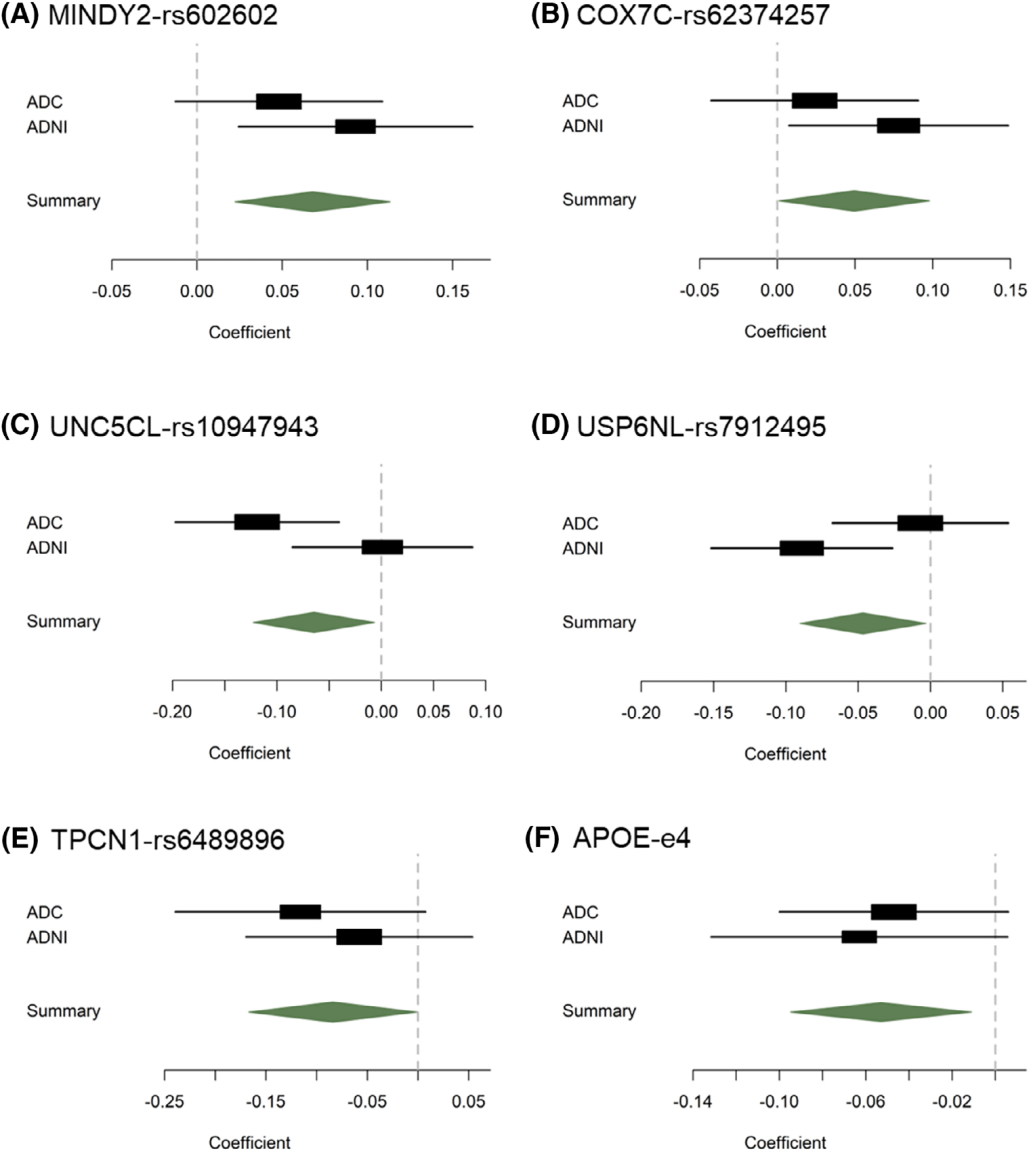
Exploratory single‐SNP level analysis of resilience and AD risk genes. Coefficients of fixed effect meta‐analysis results, as well as cohort‐specific analyses. Significance is reported for the meta‐analysis, albeit none of the results survive false discovery rate correction. Genetic variant rs602602 around gene *MINDY2* (A, *p* < 0.01) and variant rs62374257 around gene COX7C (B, *p* = 0.045) associated with higher resilience. Genetic variants rs10947943 around gene *UNC5CL* (C, P*p* = 0.029), rs7912495 around gene *USP6NL* (D, *p* = 0.036), rs6489896 around gene *TPCN1* (E, *p* = 0.047), and *APOE* ε4 (F, *p* = 0.014) associated with lower resilience. AD, Alzheimer's disease; ADC, Amsterdam Dementia Cohort; ADNI, Alzheimer's Disease Neuroimaging Initiative; *APOE*, apolipoprotein E; SNP, single nucleotide polymorphism.

The associations between cognitive resilience and AD risk were meta‐analyzed on a single‐SNP level from both cohorts (Figure 4, Figure S3 in supporting information). We found associations with lower cognitive resilience for *APOE* ε4 (β = –0.053, CI [–0.095, –0.011], *p* = 0.013), and genetic variants rs10947943 around gene *UNC5CL* (β = –0.065, CI [–0.123, –0.007], *p* = 0.029), rs7912495 around gene *USP6NL* (β = –0.047, CI [–0.09, –0.003], *p* = 0.036), and rs6489896 around gene *TPCN1* (β = –0.084, CI [–0.167, –0.001], *p* = 0.047). In addition, there were two genetic variants that were associated with higher levels of cognitive resilience. These were genetic loci rs62374257 around gene *COX7C* (β = 0.049, CI [0.001, 0.098], *p* = 0.045), and rs602602 around gene *MINDY2* (β = 0.068, CI [0.022, 0.113], *p* < 0.01). None of the presented associations on a single‐SNP level survived multiple testing correction (see Table S2 in supporting information).

### 3.5 Sensitivity analyses

When using interaction analyses to caprture resilience, we found that education modulated the relationship between cognition and brain pathology as measured by MRI in both ADC (β = –0.041, CI [–0.082, 0.000], *p* < 0.05) and ADNI (β = 0.051, CI [0.008, 0.095], *p* < 0.05), albeit in opposite directions. We found no interaction effect between MRI and *APOE* ε4 (ADC: β = 0.000, CI [–0.053, 0.054], *p* = 0.992; ADNI: β = –0.035, CI [–0.101, 0.303], *p* = 0.290) nor *APOE* ε2 (ADC: β = –0.013, CI [–0.180, 0.155], *p* = 0.883; ADNI: β = –0.017, CI [–0.237, 0.203], *p* = 0.881). Importantly, in our single‐SNP meta‐analysis, we found all six SNPs identified in our main analysis as significantly moderating the relationship between MRI and cognition (*APOE* ε4: β = –0.054, CI [–0.096, –0.012], *p* = 0.012; *UNC5CL*: β = –0.061, CI [–0.118, –0.004], *p* = 0.037; *USP6NL*: β = –0.047, CI [–0.09, –0.003], *p* = 0.036; *TPCN1*: β = –0.084, CI [–0.166, –0.001], *p* = 0.048; *COX7C*: β = 0.050, CI [0.002, 0.099], *p* = 0.041; *MINDY2*: β = 0.067, CI [0.022, 0.113], *p* = 0.004).

## 4 DISCUSSION

The current study provides a novel approach to quantifying cognitive resilience by using a CCA model on cognition and neuroimaging data. We showed its comparability across two independent cohorts and demonstrated concept validity by observing an association between our resilience framework and education, a well‐known proxy for cognitive resilience, across both cohorts. Moreover, our results suggest a potential link between *APOE* ε4, a major risk gene for developing AD, and lower cognitive resilience. As expected, no association was found between *APOE* ε2 and cognitive resilience in either cohort. Finally, in an exploratory gene‐based test, we investigated the association of 83 genetic loci previously associated with AD in the context of amyloid positivity with our operationalization of cognitive resilience. While we observed some interesting resilience–gene associations, there were no single‐SNP associations that survived multiple‐comparison correction (false discovery rate < 0.05). Altogether, the CCA models holds promise for discovery of novel genetic markers of resilience in future large‐scale multicenter GWAS.

### 4.1 CCA

We chose to employ a CCA as opposed to more traditional approaches to quantifying cognitive resilience, as it offers a valuable advantage of allowing one to incorporate varying high‐dimensional data per cohort rather than requiring variables to be similar across cohorts. This is especially useful in the context of larger studies such as GWAS analyses, in which cognitive resilience is to be defined across multiple cohorts. In this study we showed comparably high correlations of the first mode, that is, the first set of canonical variates, in both cohorts, with relatively high co‐variance explained. Further, we observed comparable loadings of the original variables on the canonical variates based on visual assessment for each domain across cohorts. This comparability in loadings was especially expected in the brain domain given that our input variables for brain were FreeSurfer‐based in both cohorts and thus the same variables. For cognition, we also expected similar cognitive domains to weigh similarly, while assuming some greater variability due to different tests. Moreover, we were able to cover one more cognitive domain in ADNI compared to ADC (i.e., the visuospatial domain). In addition to different input variables, differences in loading weights are likely to also be the result of different cohort composition.

### 4.2 Higher education associates robustly with higher resilience

The positive relationship of educational attainment and cognitive function in older age in the context of AD pathology has been extensively demonstrated.^2^, ^49^, ^50^ Specifically, it has been shown repeatedly that higher educated individuals, as measured in years of formal education, had less cognitive impairment than individuals with lower levels of education at similar levels of brain pathology.^2^, ^49^, ^50^, ^51^, ^52^ In addition, a recent study on healthy brain aging in Latin America showed that education is the most important (potentially modifiable) factor contributing to cognitive decline, demonstrating its significance over other variables such as sex and age.^53^ Therefore, education is considered an established proxy measure of cognitive resilience, highlighting the advantages in cognitive function that are not explained by the degree of brain atrophy volume or pathology.^49^, ^51^, ^52^ We successfully showed this positive relationship between education and the CCA‐based cognitive resilience framework in the two independent cohorts. In addition, our sensitivity analyses using interactions to capture resilience showed a modifying role of education in the relationship between brain pathology and cognition, albeit surprisingly with opposing directionality in ADC and ADNI.

### 4.3 *APOE* ε4 carriership associated with lower cognitive resilience

Investigating the main risk factor for sporadic AD, our meta‐analysis demonstrated an association between *APOE* ε4 carriership and lower cognitive resilience, as well as a significant role of *APOE* ε4 in moderating the relationship between brain pathology and cognition (albeit not surviving multiple testing correction). This finding was supported by a non‐significant association of *APOE* ε4 carriership and lower cognitive resilience in each cohort seperately, whereas we found no interaction effect on a single cohort level. Previous studies investigating resilience in the context of *APOE* ε4 have predominately focused on those individuals that demonstrated high rather than low cognitive resilience, highlighting possible risk mediation through modifiers, including genetic interactions with protective loci (i.e., *APOE* ε2/ε4 carriership) or modifiable risk factors (i.e., education).^54^ This is presumably due to the fact that an association between *APOE* ε4 carriership and lower cognitive resilience is in line with heightened risk to developing AD.^6^ As such, under consideration of our sensitivity analyses, our results suggest two possibilities in which (1) *APOE* ε4 carriership increases vulnerability through lower levels of cognitive resilience that otherwise might protect against cognitive decline or (2) *APOE* ε4 affects cognition independently of neurodegeneration measurable on MRI.

*APOE* ε2, on the other hand, is a key genetic protective factor in the context of AD. Our meta‐analysis did not show a significant main or interaction effect for *APOE* ε2, and we found no association of *APOE* ε2 and cognitive resilience in either cohort. While the mechanisms behind *APOE* ε2's protective effects are not fully understood, its role in reducing AD risk and slowing cognitive decline is thought to be linked primarily to its impact on cortical Aβ deposition.^54^, ^55^, ^56^ Therefore, the lack of a relationship between *APOE* ε2 carriership and cognitive resilience in our amyloid‐positive population is not surprising. However, Aβ deposition and cognitive resilience are not mutually exclusive. Other studies suggest that *APOE* ε2 may reduce AD risk through Aβ‐independent mechanisms, such as preserving gray matter volume and brain integrity in regions associated with cognitive resilience.^57^ Additionally, the *APOE* ε3 Christchurch mutation, another *APOE* variant, has been linked to relative protection from AD.^8^ For instance, a homozygous carrier of the *APOE* ε3 Christchurch variant developed MCI nearly three decades later than expected, despite AD‐causative *PSEN1* carriership and presence of amyloidosis.^58^ Distinguishing between protection from AD pathology and cognitive resilience despite brain pathology is hence crucial.

### 4.4 Some genetic loci associated with AD risk show potential involvement in cognitive resilience

In addition to the major risk and protective variants of *APOE*, the most influential genetic factor in the context of AD, a number of other genetic loci have been found to be significantly associated with risk of developing AD.^5^ Among these genetic loci, genetic variants around genes *UNC5CL*, *USP6NL*, and *TPCN1* were associated with lower cognitive resilience in our study, and were found to moderate the relationship between brain pathology and cognition, suggesting a greater vulnerability to AD. In addition, two SNPs were associated with higher levels of cognitive resilience. All effects were confirmed in the interaction resilience analysis, highlighting the potential role of both variants in enhancing resilience. Of these, variant rs62374257 is located around gene *COX7C*, which contributes to mitochondrial bioenergetics, suggesting a possible involvement of brain metabolic processes in resilience to AD.^59^ The second SNP associated with high cognitive resilience, rs602602, is located near gene *MINDY2*. This association stands in contrast to another study that reported a positive association of *MINDY2* and the expression of the AD‐causative *APP* gene in the risk of developing AD.^60^ However, none of the aforementioned genetic loci surived a correction for multiple testing, thus warranting careful interpretation of the results.

### 4.5 Strengths and limitations

This study has several strengths, including the application of a well‐established startistical method in a novel context across two independent cohorts. We were able to show that the first mode of the CCA seems to capture similar modes across different cohorts, yielding a major methodological advantage for multi‐cohort studies that so far were limited by composite measures or similar tests. There are also several limitations to consider. Despite key advantages regarding the application of a CCA compared to the standard residual approach in terms of capturing a more well‐rounded and cohort‐specific definition of resilience through use of all data available, this method does not solve known methodological drawbacks of residual approaches.^15^ As such, we propose using CCAs over standard residual approaches as a better way of residualizing, yet recognize the need for future research focusing on developing different methods of measuring resilience that do not suffer the inherent limitations of residuals.^15^ Moreover, in this study, we tested associations of key genetic risk and protective factors of AD, both of these loci being located on the *APOE* gene (alleles ε4 and ε2, respectively). Despite these genes being widely studied in the context of AD, it is important to acknowledge that these loci do not necessarily relate to cognitive resilience to AD to the same extent, or may not be related to resilience at all. Instead, *APOE* might affect cognition in the context of AD only through brain resistance, rather than resilience. Furthermore, we believe that cognitive resilience is a complex trait influenced by many genes, each contributing only a small effect. As a result, detecting these genes necessitates a larger sample size than what is available in this study. Future studies should thus investigate the relationship between cognitive resilience to AD and AD‐related genes in unbiased GWAS studies. Another aspect to consider when interpreting the results of this study is that both cohorts were highly educated and predominantly White. This should be addressed in future investigations through inclusion of racially/ethnically diverse cohorts and individuals with lower socioeconomic status. In addition, the use of brain atrophy as a proxy for pathology, while practical due to the availability of large datasets, is inherently imperfect. Future research should prioritize more specific biomarkers, such as plasma phosphorylated tau217, tau PET imaging, or downstream markers in biofluids to provide a more precise understanding of resilience mechanisms. Altogether, this research serves as a proof of concept for future, more extensive GWAS studies, which could provide additional insights into the polygenic nature of cognitive resilience and help identify novel genetic contributors.

## CONFLICT OF INTEREST STATEMENT

Frederik Barkhof is a steering committee or data safety monitoring board member for Biogen, Merck, Eisai and Prothena, advisory board member for Combinostics, Scottish Brain Sciences, Alzheimer Europe. He is a consultant for Roche, Celltrion, Rewind Therapeutics, Merck, Bracco, and has research agreements with ADDI, Merck, Biogen, GE Healthcare, Roche. Finally, he is co‐founder and shareholder of Queen Square Analytics LTD. Jacob Vogel has received consultancy/speaker fees from Manifest Technologies, Inc. Wiesje M. van der Flier has been an invited speaker at Biogen MA Inc, Danone, Eisai, WebMD Neurology (Medscape), NovoNordisk, Springer Healthcare, European Brain Council, and she participated in advisory boards of Biogen MA Inc, Roche, and Eli Lilly. She is a member of the steering committee of EVOKE/EVOKE+ (NovoNordisk). All funding is paid to her institution. She is consultant to Oxford Health Policy Forum CIC, Roche, Biogen MA Inc, and Eisai. All funding is paid to her institution. She is member of the steering committee of PAVE, and Think Brain Health, was associate editor of *Alzheimer's Research & Therapy* in 2020/2021, and currently is associate editor at *Brain*, and is member of Supervisory Board (Raad van Toezicht) Trimbos Instituut. Rik Ossenkoppele has received research funding/support from  Avid Radiopharmaceuticals, Janssen Research & Development, Roche, Quanterix, and Optina Diagnostics; has given lectures in symposia sponsored by GE Healthcare; received speaker fees from Springer; is an advisory board member for Asceneuron and a steering committee member for Biogen and Bristol Myers Squibb. All the aforementioned has been paid to his institutions. He is an editorial board member of *Alzheimer's Research & Therapy* and the *European Journal of Nuclear Medicine and Molecular Imaging*. The other authors have no conflicts of interest to declare. Author disclosures are available in the supporting information.

## Supporting information



Supporting Information

Supporting Information
