# Supplementary material for: A novel approach to resilience and its links with education and Alzheimer's disease genetics
Source: Alzheimers Dement. 2025 Jul 4;21(7):e70379. doi: 10.1002/alz.70379 (PMC12231230; doi:10.1002/alz.70379)

**Supplemental Table 1. Conversion of the qualitative Verhage scale for educational attainment to years of education**

| **Verhage score** | **Description** | **Years of education** |
| --- | --- | --- |
| **1** | <6 years of primary education | <6 (elementary school) |
| **2** | Completed 6 years of primary education | 6 |
| **3** | >6 years of primary education without completed specialized education | 8 |
| **4** | Completed higher education below junior high school level (“MULO”) | 9 |
| **5** | Junior high school level (“MULO”, “MAVO”, “VMBO”) | 10–11 |
| **6** | High school/community college level (“HBS”, “HAVO”, “VWO”, “HBO”) | 12–18 |
| **7** | College level | >18 (university) |

**Supplemental Table 2. Single-SNP level meta-analysis**

| **SNP** | **Beta** | **SE** | **95% CI** | **p-value** | **pFDR** | **direction** |  |
| --- | --- | --- | --- | --- | --- | --- | --- |
| ABCA1-rs1800978 | -0.028 | 0.030 | [ -0.087 - 0.031 ] | 0.350 | 0.886 | -- |  |
| ABCA7-rs12151021 | 0.019 | 0.022 | [ -0.025 - 0.062 ] | 0.398 | 0.886 | +- |  |
| ABI3-rs616338 | 0.044 | 0.086 | [ -0.124 - 0.212 ] | 0.607 | 0.937 | +- |  |
| ACE-rs4277405 | 0.006 | 0.023 | [ -0.039 - 0.05 ] | 0.807 | 0.955 | +- |  |
| ADAM17-rs72777026 | -0.003 | 0.031 | [ -0.063 - 0.057 ] | 0.928 | 0.985 | +- |  |
| ADAMTS1-rs2830489 | -0.020 | 0.024 | [ -0.067 - 0.028 ] | 0.415 | 0.886 | =- |  |
| ANK3-rs7068231 | -0.022 | 0.022 | [ -0.064 - 0.021 ] | 0.322 | 0.886 | -- |  |
| ANKH-rs112403360 | 0.001 | 0.040 | [ -0.077 - 0.079 ] | 0.976 | 0.985 | +- |  |
| APH1B-rs117618017 | 0.047 | 0.032 | [ -0.017 - 0.11 ] | 0.149 | 0.839 | ++ |  |
| APOE2-APOE2 | 0.001 | 0.066 | [ -0.127 - 0.13 ] | 0.985 | 0.985 | -+ |  |
| APOE4-APOE4 | -0.053 | 0.021 | [ -0.095 - -0.011 ] | **0.013** | 0.555 | -- |  |
| APP-rs2154481 | 0.008 | 0.021 | [ -0.033 - 0.049 ] | 0.703 | 0.937 | -+ |  |
| BCKDK-rs889555 | -0.017 | 0.024 | [ -0.064 - 0.031 ] | 0.493 | 0.886 | -+ |  |
| BIN1-rs6733839 | 0.007 | 0.021 | [ -0.035 - 0.049 ] | 0.739 | 0.937 | -+ |  |
| BLNK-rs6584063 | 0.021 | 0.055 | [ -0.088 - 0.129 ] | 0.706 | 0.937 | ++ |  |
| CASS4-rs6014724 | -0.050 | 0.039 | [ -0.127 - 0.026 ] | 0.199 | 0.886 | -- |  |
| CD2AP-rs7767350 | -0.028 | 0.023 | [ -0.073 - 0.017 ] | 0.221 | 0.886 | -- |  |
| CLNK-rs6846529 | 0.013 | 0.023 | [ -0.033 - 0.058 ] | 0.587 | 0.931 | +- |  |
| CLU-rs11787077 | 0.017 | 0.022 | [ -0.026 - 0.06 ] | 0.435 | 0.886 | ++ |  |
| COX7C-rs62374257 | 0.049 | 0.025 | [ 0.001 - 0.098 ] | **0.045** | 0.652 | ++ |  |
| CR1-rs679515 | -0.027 | 0.026 | [ -0.078 - 0.023 ] | 0.290 | 0.886 | +- |  |
| CTSB-rs1065712 | 0.032 | 0.045 | [ -0.056 - 0.121 ] | 0.475 | 0.886 | ++ |  |
| CTSH-rs12592898 | 0.021 | 0.031 | [ -0.041 - 0.083 ] | 0.505 | 0.886 | +- |  |
| DOC2A-rs1140239 | -0.021 | 0.022 | [ -0.064 - 0.023 ] | 0.359 | 0.886 | -- |  |
| EED-rs3851179 | 0.019 | 0.022 | [ -0.024 - 0.062 ] | 0.392 | 0.886 | +- |  |
| EPDR1-rs6966331 | -0.015 | 0.022 | [ -0.058 - 0.029 ] | 0.509 | 0.886 | +- |  |
| EPHA1-rs11771145 | -0.027 | 0.022 | [ -0.071 - 0.016 ] | 0.217 | 0.886 | -- |  |
| FERMT2-rs17125924 | 0.011 | 0.034 | [ -0.055 - 0.077 ] | 0.747 | 0.937 | +- |  |
| FOXF1-rs16941239 | -0.029 | 0.071 | [ -0.168 - 0.11 ] | 0.685 | 0.937 | +- |  |
| GRN-rs5848 | -0.005 | 0.024 | [ -0.051 - 0.041 ] | 0.838 | 0.976 | -+ |  |
| HLA-DQA1-rs6605556 | 0.011 | 0.032 | [ -0.051 - 0.073 ] | 0.725 | 0.937 | +- |  |
| HS3STS-rs785129 | 0.001 | 0.022 | [ -0.043 - 0.045 ] | 0.978 | 0.985 | +- |  |
| ICA1-rs10952097 | 0.010 | 0.037 | [ -0.063 - 0.083 ] | 0.786 | 0.944 | +- |  |
| IDUA-rs3822030 | 0.022 | 0.021 | [ -0.02 - 0.063 ] | 0.313 | 0.886 | +- |  |
| IGH gene cluster-rs10131280 | 0.043 | 0.038 | [ -0.031 - 0.117 ] | 0.251 | 0.886 | ++ |  |
| IGH gene cluster-rs7157106 | -0.024 | 0.032 | [ -0.087 - 0.04 ] | 0.463 | 0.886 | -+ |  |
| IL34-rs4985556 | -0.024 | 0.033 | [ -0.089 - 0.041 ] | 0.472 | 0.886 | -- |  |
| INPP5D-rs10933431 | 0.016 | 0.026 | [ -0.035 - 0.068 ] | 0.532 | 0.893 | ++ |  |
| JAZF1-rs1160871 | 0.002 | 0.025 | [ -0.048 - 0.052 ] | 0.930 | 0.985 | -+ |  |
| KLF16-rs149080927 | -0.037 | 0.023 | [ -0.082 - 0.008 ] | 0.104 | 0.795 | -- |  |
| LILRB2-rs587709 | 0.008 | 0.024 | [ -0.039 - 0.056 ] | 0.731 | 0.937 | ++ |  |
| MAF-rs450674 | 0.008 | 0.022 | [ -0.036 - 0.051 ] | 0.731 | 0.937 | -+ |  |
| MAPT-rs199515 | 0.039 | 0.026 | [ -0.012 - 0.09 ] | 0.132 | 0.839 | ++ |  |
| MINDY2-rs602602 | 0.068 | 0.023 | [ 0.022 - 0.113 ] | **0.003** | 0.293 | ++ |  |
| MME-rs16824536 | -0.019 | 0.049 | [ -0.115 - 0.077 ] | 0.698 | 0.937 | -+ |  |
| MME-rs61762319 | -0.058 | 0.066 | [ -0.188 - 0.071 ] | 0.378 | 0.886 | -- |  |
| MS4A4A-rs1582763 | 0.003 | 0.022 | [ -0.04 - 0.047 ] | 0.889 | 0.985 | +- |  |
| MYO15A-rs2242595 | 0.027 | 0.034 | [ -0.039 - 0.092 ] | 0.427 | 0.886 | ++ |  |
| NCK2-rs143080277 | 0.083 | 0.146 | [ -0.203 - 0.37 ] | 0.569 | 0.920 | +- |  |
| PLCG2-rs12446759 | -0.020 | 0.022 | [ -0.064 - 0.023 ] | 0.363 | 0.886 | -- |  |
| PLCG2-rs72824905 | 0.081 | 0.124 | [ -0.163 - 0.325 ] | 0.517 | 0.886 | +- |  |
| PLEKHA1-rs7908662 | -0.015 | 0.022 | [ -0.058 - 0.027 ] | 0.477 | 0.886 | -+ |  |
| PRKD3-rs17020490 | -0.031 | 0.030 | [ -0.091 - 0.029 ] | 0.307 | 0.886 | -- |  |
| PTK2B-rs73223431 | 0.015 | 0.021 | [ -0.027 - 0.057 ] | 0.490 | 0.886 | +- |  |
| RASGEF1C-rs113706587 | -0.010 | 0.037 | [ -0.083 - 0.063 ] | 0.783 | 0.944 | +- |  |
| RBCK1-rs1358782 | 0.038 | 0.026 | [ -0.014 - 0.089 ] | 0.152 | 0.839 | ++ |  |
| RHOH-rs2245466 | -0.041 | 0.024 | [ -0.088 - 0.006 ] | 0.085 | 0.772 | -- |  |
| SCIMP-rs7225151 | -0.052 | 0.031 | [ -0.113 - 0.008 ] | 0.091 | 0.772 | -- |  |
| SEC61G-rs76928645 | 0.055 | 0.036 | [ -0.016 - 0.127 ] | 0.128 | 0.839 | -+ |  |
| SHARPIN-rs34173062 | -0.018 | 0.040 | [ -0.096 - 0.06 ] | 0.652 | 0.937 | -= |  |
| SIGLEC11-rs9304690 | -0.044 | 0.025 | [ -0.092 - 0.005 ] | 0.076 | 0.772 | -- |  |
| SLC24A4-rs12590654 | 0.017 | 0.023 | [ -0.028 - 0.063 ] | 0.450 | 0.886 | +- |  |
| SLC24A4-rs7401792 | -0.001 | 0.022 | [ -0.045 - 0.043 ] | 0.958 | 0.985 | -+ |  |
| SLC2A4RG-rs6742 | -0.009 | 0.027 | [ -0.062 - 0.044 ] | 0.741 | 0.937 | -+ |  |
| SNX1-rs3848143 | -0.011 | 0.026 | [ -0.062 - 0.04 ] | 0.670 | 0.937 | +- |  |
| SORL1-rs11218343 | -0.004 | 0.057 | [ -0.116 - 0.108 ] | 0.944 | 0.985 | -= |  |
| SORL1-rs74685827 | 0.144 | 0.085 | [ -0.023 - 0.311 ] | 0.092 | 0.772 | ++ |  |
| SORT1-rs141749679 | 0.028 | 0.153 | [ -0.272 - 0.329 ] | 0.854 | 0.976 | +- |  |
| SPDYE3-rs7384878 | 0.004 | 0.023 | [ -0.042 - 0.05 ] | 0.860 | 0.976 | ++ |  |
| SPI1-rs10437655 | 0.028 | 0.022 | [ -0.015 - 0.07 ] | 0.204 | 0.886 | ++ |  |
| SPPL2A-rs8025980 | -0.023 | 0.022 | [ -0.067 - 0.021 ] | 0.299 | 0.886 | -- |  |
| TMEM106B-rs13237518 | 0.006 | 0.021 | [ -0.035 - 0.048 ] | 0.758 | 0.937 | +- |  |
| TNIP1-rs871269 | 0.022 | 0.023 | [ -0.023 - 0.067 ] | 0.334 | 0.886 | ++ |  |
| TPCN1-rs6489896 | -0.084 | 0.042 | [ -0.167 - -0.001 ] | **0.047** | 0.652 | -- |  |
| TREM2-rs143332484 | 0.111 | 0.079 | [ -0.044 - 0.265 ] | 0.160 | 0.839 | ++ |  |
| TREM2-rs75932628 | -0.076 | 0.127 | [ -0.324 - 0.172 ] | 0.550 | 0.905 | +- |  |
| TREML2-rs60755019 | 0.214 | 0.205 | [ -0.188 - 0.616 ] | 0.297 | 0.886 | ++ |  |
| TSPAN14-rs6586028 | -0.033 | 0.028 | [ -0.088 - 0.022 ] | 0.239 | 0.886 | -- |  |
| TSPOAP1-rs2526377 | 0.018 | 0.021 | [ -0.024 - 0.06 ] | 0.404 | 0.886 | ++ |  |
| UMAD1-rs6943429 | 0.001 | 0.021 | [ -0.042 - 0.043 ] | 0.983 | 0.985 | -+ |  |
| UNC5CL-rs10947943 | -0.065 | 0.030 | [ -0.123 - -0.007 ] | **0.029** | 0.652 | -+ |  |
| USP6NL-rs7912495 | -0.047 | 0.022 | [ -0.09 - -0.003 ] | **0.036** | 0.652 | -- |  |
| WDR12-rs139643391 | 0.002 | 0.033 | [ -0.062 - 0.066 ] | 0.945 | 0.985 | +- |  |
| WDR81-rs35048651 | -0.010 | 0.026 | [ -0.062 - 0.041 ] | 0.700 | 0.937 | -- |  |
| *In ADNI, rs117618017 and rs7157106 were replaced with rs75763893 and rs2753568, respectively. | | | | | | | |

**Supplemental Figure 1. CCA model in ADNI cohort**


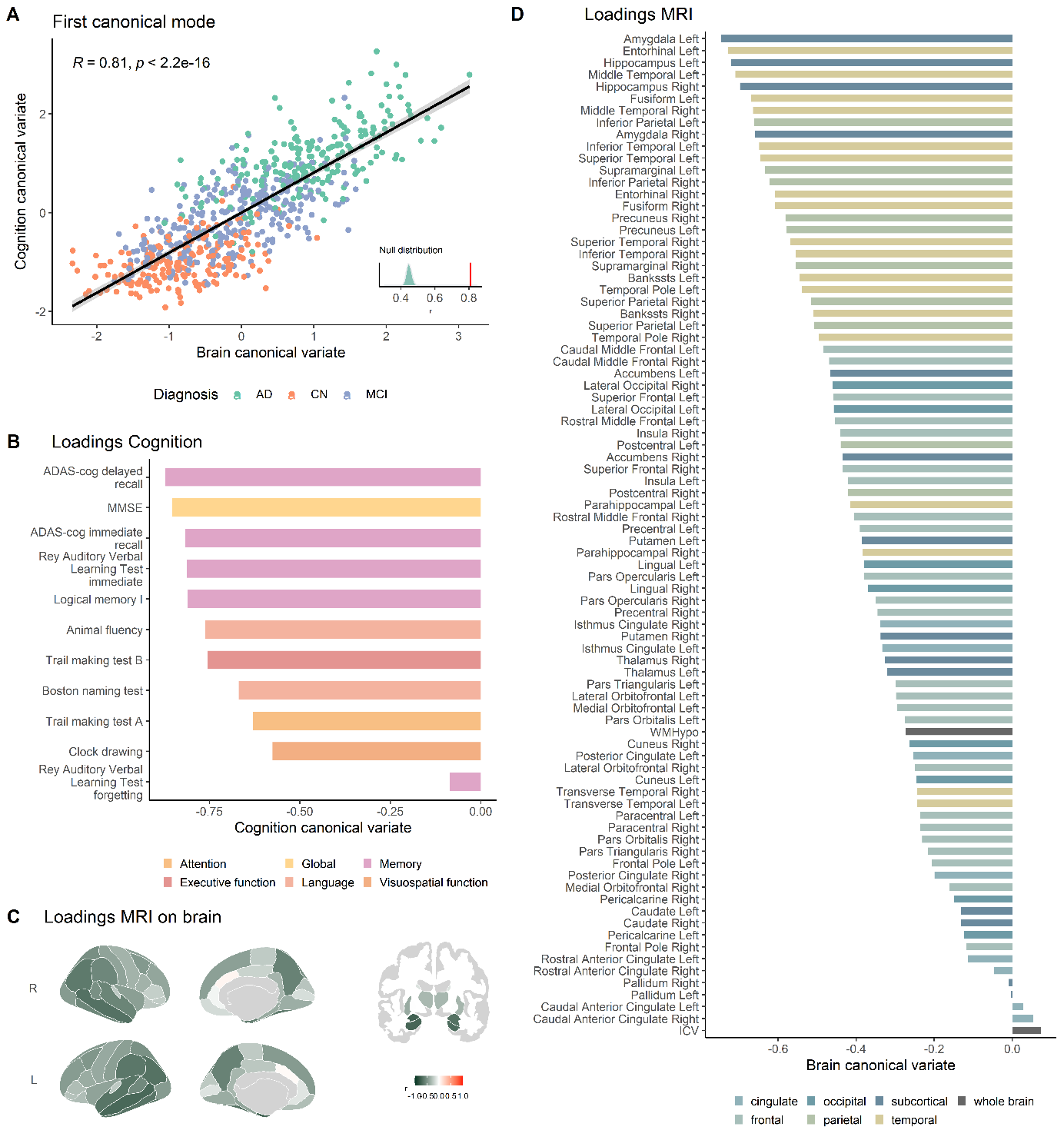


**Supplemental Figure 2. Correlation of MRI variable loadings on X canonical variate between ADC and ADNI cohorts**


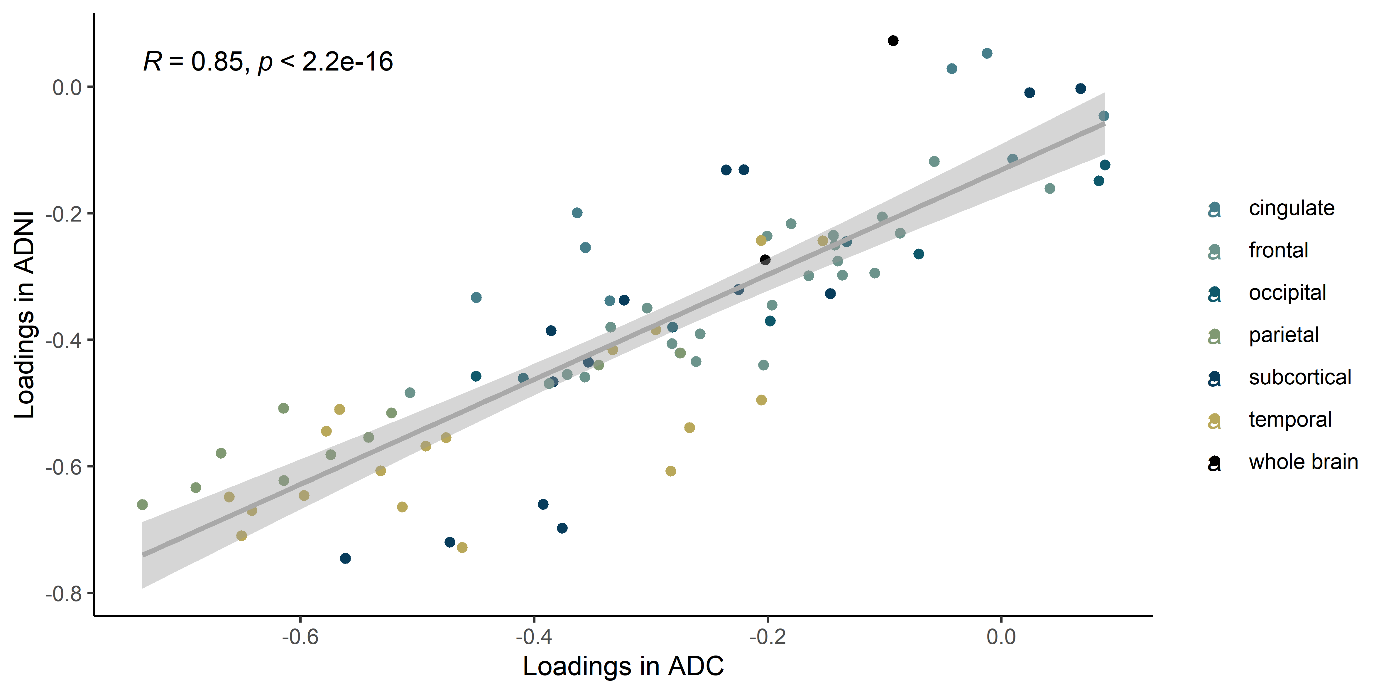


**Supplemental Figure 3. Single-SNP effects within each cohort**


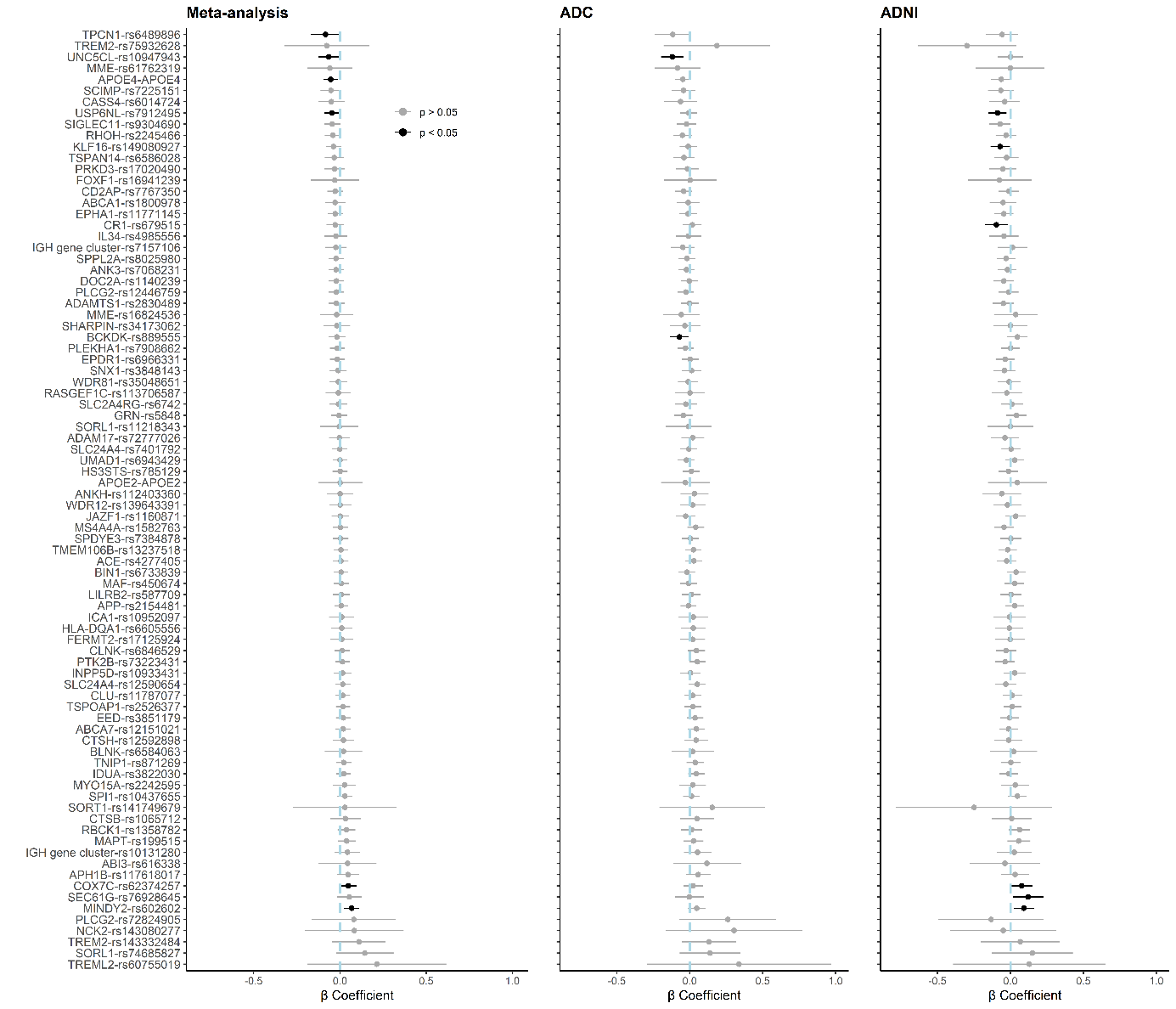

Supplement: Supplementary file 2 — Supporting Information [file ALZ-21-e70379-s002.docx]
